# Metabolic Reprogramming and Reactive Oxygen Species in T Cell Immunity

**DOI:** 10.3389/fimmu.2021.652687

**Published:** 2021-03-31

**Authors:** Hao-Yun Peng, Jason Lucavs, Darby Ballard, Jugal Kishore Das, Anil Kumar, Liqing Wang, Yijie Ren, Xiaofang Xiong, Jianxun Song

**Affiliations:** ^1^ Department of Microbial Pathogenesis and Immunology, Texas A&M University Health Science Center, Bryan, TX, United States; ^2^ Department of Biochemistry and Biophysics, Texas A&M University, College Station, TX, United States

**Keywords:** T cells, cell metabolism, reactive oxygen species, immunity, disease

## Abstract

T cells undergo metabolic reprogramming and multiple biological processes to satisfy their energetic and biosynthetic demands throughout their lifespan. Several of these metabolic pathways result in the generation of reactive oxygen species (ROS). The imbalance between ROS generation and scavenging could result in severe damage to the cells and potential cell death, ultimately leading to T cell-related diseases. Interestingly, ROS play an essential role in T cell immunity. Here, we introduce the important connectivity between T cell lifespan and the metabolic reprogramming among distinct T cell subsets. We also discuss the generation and sources of ROS production within T cell immunity as well as highlight recent research concerning the effects of ROS on T cell activities.

## Introduction

The rapid invasion and spreading of foreign pathogens often catch our immune system off guard. As critical host-mediated immune cells, T cells must be rapidly responded to foreign substances and efficiently proliferate in a timely manner. To grow, proliferate, and differentiate, T cells undergo metabolic reprogramming to meet their bioenergetic needs. T cells can engage a variety of distinct metabolic pathways, including glycolysis, oxidative phosphorylation (OXPHOS), fatty acid synthesis, etc. Energy production from those pathways unavoidably generates reactive oxygen species (ROS), which cause damage to the cell. However, there is compelling evidence that ROS acts as a critical signaling component in T cell immunity. In the following sections, we will highlight the metabolic reprogramming of distinct T cell subsets, including thymocytes, naive T cells, effector T cells, differentiated T cells, and memory T cells. We will address the sites and sources of ROS production in T cells, as well as the emerging concepts surrounding the impact of ROS production on T cell development, activation, differentiation, and apoptosis.

## Metabolic Reprogramming of Various T Cell Subsets

T cells originate from bone marrow and mature in the thymus. While maturing in the thymus, thymocytes encounter steps of selection to ensure the generation of mature T cells with the following characteristics: “foreign” antigen recognition, self-antigen tolerization, and accurate surface marker expression to perform effector functions. It is estimated that 95–97% of thymocytes are eliminated due to not meeting these criteria (1). During the early stage of thymic maturation, the glucose transporters Glut1 and Glut4 are highly expressed, suggesting an increase in glycolysis (2–4). The expression of Glut1 and Glut4 is significantly reduced as thymocytes mature to a later stage (2–4). The mature thymocytes, called naive T (Tn) cells, leave the thymus, circulate into the bloodstream, and finally arrive at the secondary lymphoid tissues, such as the spleen and lymph nodes (LNs). Peripheral naive T cells remain in quiescence and only accumulate essential cellular building blocks (6, 7). Tn cells generate the minimal energy needed to function by metabolizing glucose to pyruvate. The pyruvate will then enter the tricarboxylic acid cycle (TCA cycle) and undergo oxidative phosphorylation (OXPHOS) (6, 8). Alternatively, Tn may also utilize fatty acid synthesis (FAO) to produce sufficient ATP levels (8).

Tn cells are activated by the binding of the T cell receptors (TCR) and the antigen peptides on the major histocompatibility complex (MHC) from antigen-presenting cells (APC) with the help of costimulatory molecules (9). This ligation further triggers multiple signaling pathways, and the activated T cells expand and transform into effector T (Teff) cells with the assistance of multiple cytokines. To meet the demand for rapid proliferation, clonal expansion, and effector functions, Teff cells shift from mainly conducting OXPHOS to aerobic glycolysis (7, 10–14). Many studies have shown that glutaminolysis, pentose phosphate pathway, lipid synthesis, and OXPHOS are enhanced in Teff cells as well (10, 12, 13).

**Figure 1:**
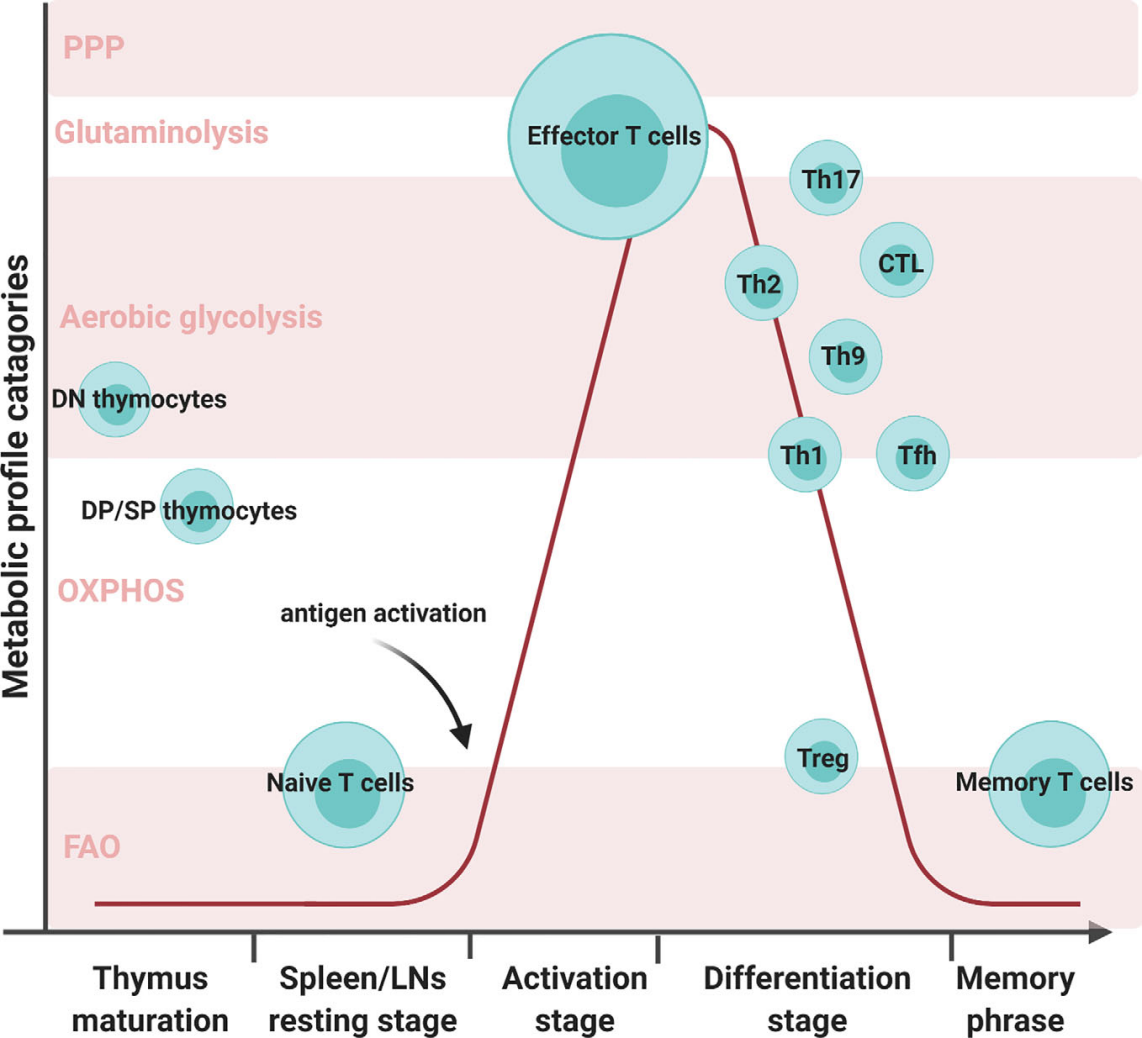
Various T cell subsets development with metabolic reprogramming status. There are five metabolic profile categories: the pentose phosphate pathway, glutaminolysis, aerobic glycolysis, oxidative phosphorylation (OXPHOS), and fatty acid oxidation (FAO). Different T cell subsets alter their metabolic status at different developmental stages. Double negative (DN) cells are the initial stage of thymocytes. These cells will mainly use aerobic glycolysis during proliferation. In later stages of thymocyte development, double-positive (DP) cells and single CD4 or CD8 T cells mature and prepare to migrate through the bloodstream to the secondary organs. During this stage, the matured thymocytes preferentially utilize OXPHOS and FAO to meet their metabolic needs. Naive T cells in spleens and LNs continue in quiescence as thymocytes to minimize energy consumption. Once encountering antigens, T cells activate and proliferate to face foreign assailants. In order to combat foreign pathogens, effector T cells transition their metabolism from OXPHOS to aerobic glycolysis. T cells will progress into the differentiation stage where there are multiple T-cell subsets, such as T helper 1 (Th1), T helper 2 (Th2), T helper 9 (Th9), T helper 17 (Th17), regulatory T (Treg), and T follicular B helper (Tfh) cells. Although all T cell subsets utilize aerobic glycolysis, the varying subsets employ different metabolic processes. Th17 can utilize glutaminolysis and both Th1 and Tfh can conduct OXPHOS in addition to aerobic glycolysis. Memory T cells exhibited both OXPHOS and FAO to maintain their function.

CD8^+^ Teff cells produce large amounts of perforin and granzyme B to eliminate foreign pathogens, viral-infected cells, and tumor cells (15). In comparison, CD4^+^ Teff cells secrete an array of cytokines and recruit other immune cells. CD4^+^ Teff cells differentiate into functionally distinct subsets in different cytokine environment: Th1, Th2, Th9, Th17, etc. Different subsets of differentiated Teff show distinct metabolic profiles, shown in **Figure 1**. T helper 1 (Th1) cells eliminate intracellular pathogens by producing IFNγ and can activate macrophages (16). Deficiency of lactate dehydrogenase A (LDHA) has shown reduced IFN-γ levels under T helper 1 (Th1) conditions (13). Another study mentioned that upon defective Th1 conditions, T cells fail to upregulate glycolysis and OXPHOS (17). Th1 cells utilize some OXPHOS and mainly glycolysis by expressing high levels of Glut 1 (14, 18, 19). T helper 2 (Th2) cells protect against extracellular parasites by secreting cytokine IL-4, IL-5, and IL-13 (20). Studies have shown that treatment with glycolysis inhibitor 2-deoxyglucose (2-DG) impaired Th2 differentiation (20, 21). These cells utilize the aerobic glycolytic pathway by expressing the most Glut1 than other Teff cells to meet their developmental and functional needs (14, 20). T helper 9 (Th9) cells secrete IL-9 to eliminate extracellular parasites. There have been numerous discoveries pertaining to the metabolic pathways of other Teff cells; however, how Th9 cells modulate their metabolic pathways remains unclear. A study conducted by Wang has shown that Th9 cell differentiation is dependent upon the TAK1-SIRT1-mTOR-HIF1α-glycolysis pathway (22). T helper 17 (Th17) cells protect against extracellular bacteria and fungi with an imbalance of Th17 leading to autoimmune disease (23). Various studies have shown that Th17 cells uptake glucose and undergo glutaminolysis (14, 18, 23–25). T follicular B helper T (Tfh) cells are distinguished from other Teff cells by their unique role in memory B cell development and plasma cell maturation (26). Tfh cells use both aerobic glycolysis and OXPHOS but lower levels with respect to Th1 cells (18). Regulatory T (Treg) cells have immunosuppressive capacities not seen in other T cell subsets. Unlike other T cell subsets, Treg exhibited both OXPHOS and FAO to maintain function (18, 24, 25). The surviving population of Teff cells remodels into memory T (Tm) cells which will later respond to future threats the Tm cells have previously experienced (11). Unlike high glycolytic Teff cells, Tm cells depend on OXPHOS and FAO to meet their metabolic needs (6, 7, 11, 12, 14, 27). The distinct metabolic profiling of T cell subsets may imply their biosynthetic needs and support their differing functional properties.

T cells rewire their metabolism by processing oxidative and catalytic activities to meet their demands at various points throughout their lifespan. Concomitantly, Reactive Oxygen Species (ROS), generated as a byproduct of the oxidative metabolism process, is a requisite secondary signaling factor in T cell immunity.

## Double-Edged Effect of ROS in T Cells

Reactive Oxygen Species (ROS) are a group of highly reactive, unstable radicals and non-free radical compounds containing oxygen. Examples of ROS include superoxide $\left(O_{2}^{-}\right)$, hydrogen peroxide (H_2_O_2_), singlet oxygen, ozone, peroxynitrite (ONOO^−^), and hydroxyl radical (·OH), with superoxide and hydrogen peroxide being the most common under physiological conditions (28–30). Although superoxide is the original form of ROS, it is highly unstable, and upon forming, reacts with surrounding molecules to form hydrogen peroxide, peroxynitrite, and all other ROS. Under normal conditions, ROS levels are tightly regulated by various endogenous antioxidant enzymes, including superoxide dismutase (SODs), catalases (CAT), glutathione peroxidases, and multiple antioxidant molecules, such as pyruvate, α-ketoglutarate, and glutathione (GSH) (28, 29). Low to moderate ROS levels are essential for cell survival and proliferation (28, 31, 32). When excess ROS overwhelms the antioxidant systems, oxidative stress occurs, leading to harmful effects on cellular organisms, such as inducing DNA mutations (28, 31, 33), altering lipid metabolism (31, 34), and further inducing cell death (5). Although high ROS levels result in harm to the organism, a large body of research finds that ROS acts as one of the essential secondary messengers playing a role in T cell function (35, 36).

## Sources of ROS Production in T Cells

**Figure 2:**
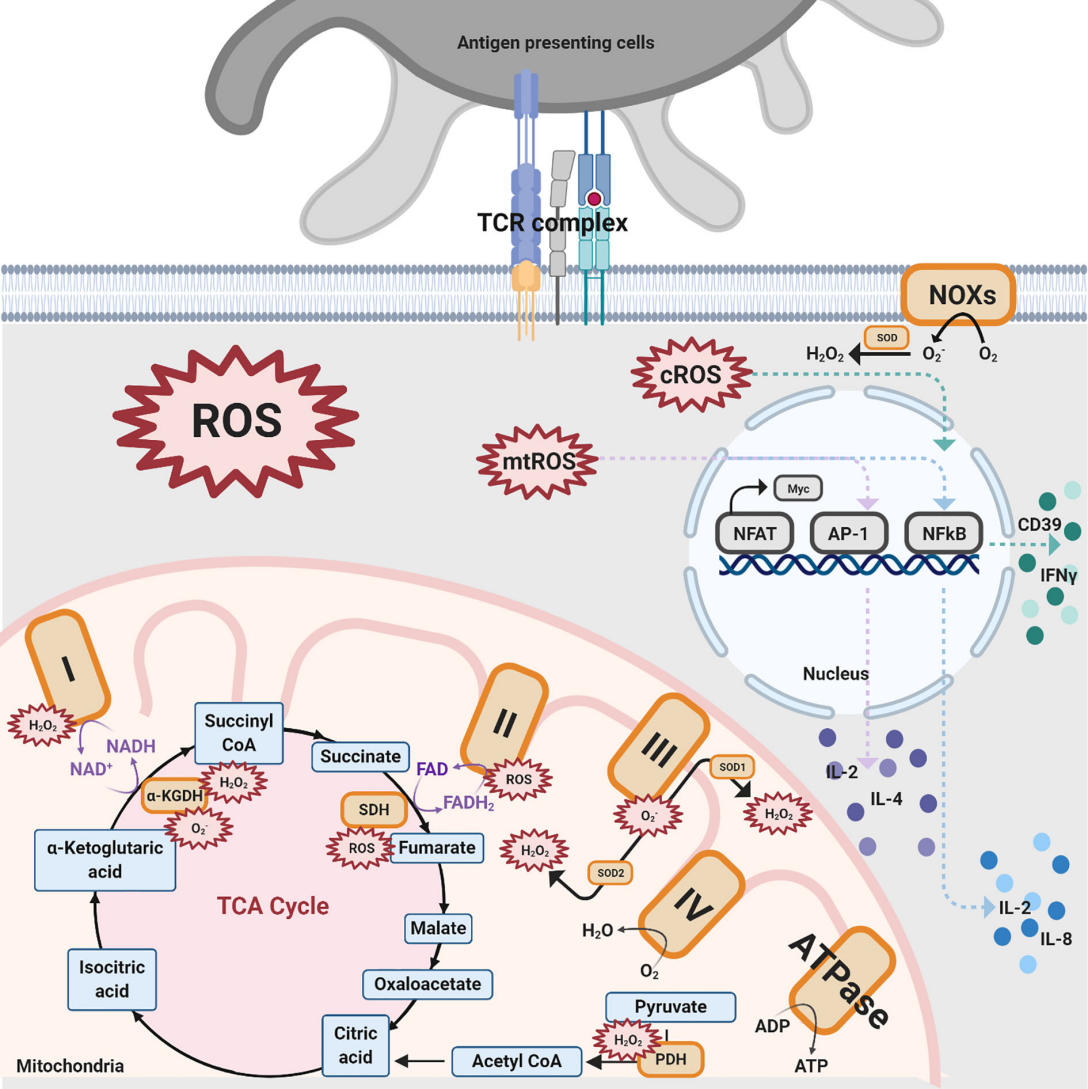
Different sources of ROS impacting T cell activation. There are three ROS generation sources mentioned in this review that impact T cell activation: NOXs, mitochondrial complexes for OXPHOS, and TCA enzymes. NOXs are a group of enzymes responsible for the transfer of electrons from oxygen to cytoplasmic superoxide. In this figure, black solid arrows indicate the production of these pathways while purple solid arrows indicate the electrons transfer reactions for coenzymes within the TCA cycle and mitochondrial complexes. Colored dashed arrows designate the inductions of transcription factors, such as NFAT, AP-1, NFkB, and further upregulate various cytokines. The orange-colored box denotes protein complexes while the compounds that are produced from the TCA cycle are shown in blue. The red spiked border indicates ROS generation.

Various sources produce ROS in T cells, with the majority of production coming from mitochondria, NADPH oxidases (NOXs), lipid metabolism, and several other enzymes, such as cyclooxygenases and others (5, 28, 35, 37–46). In this section, the two sources that produce ROS in T cells, mitochondria and NOXs, will be discussed (**Figure 2**).

### Oxidative Phosphorylation—The Primary Cell Pathway That Produces ROS in Mitochondria

Oxidative phosphorylation (OXPHOS) is one of the pathways by which ROS are generated in T cells. OXPHOS is a biological process that transports electrons, generates proton gradients, and utilizes oxygen or simple sugars to make adenosine triphosphate (ATP), the primary energy source of the cell. The multiple complexes and coenzymes of the electron transport chain (ETC) are needed to conduct OXPHOS. NADH: ubiquinone oxidoreductase (Mitochondrial complex I) transfers electrons from one of the coenzymes, defined as electron carriers, NADH to ubiquinone, which proceeds to pump the protons into the intermembrane space (47). The other coenzyme, reduced flavin adenine dinucleotide (FADH2), donates electrons through succinate dehydrogenase (Mitochondrial complex II). The electrons are then transported to ubiquinol-cytochrome c oxidoreductase (Mitochondrial complex III) and cytochrome c oxidase (Mitochondrial complex IV), where oxygen is reduced to water. During the process of electron donation, the protons within the matrix are pumped to the mitochondrial intermembrane space through complexes I, III, and IV (47). The proton gradient in the intermembrane space drives ATP synthase to produce ATP. However, OXPHOS is not a perfect mechanism. There is a 0.2–2% leakage of electrons in mitochondrial complexes I–III, primarily complexes I and III, resulting in the creation of superoxide and hydrogen peroxide (31, 47). Specifically, a total of 11 sites have been proven to generate ROS in the ETC, a finding which has been reviewed elsewhere (47, 48).

Superoxide can be generated in complex I through both directions of the reaction, meaning electron transfer from NADH to ubiquinone or reduced ubiquinone to NAD^+^ (5). When a complex I inhibitor, such as Rotenone, is introduced, the ROS production from the reverse electron transfer (RET) from NADH to ubiquinone is inhibited (40–43). There is supporting evidence highlighting that ROS production from complex II also plays an essential role despite the negligible amount produced by the complex under normal conditions (44, 45). However, the major ROS product generated from complex II is still under debate. The results from Siebels and Drose (46) support that hydrogen peroxide is the major product from complex II-generated ROS while the results from Grivennikova (44) identified superoxide as the major product. Complex III produces the second most amount of ROS where superoxide is converted into stable hydrogen peroxide by two superoxide dismutase (SOD) isoforms SOD1 in the intermembrane space and SOD2 in the matrix (31, 46).

### The TCA Cycle’s Role in Mitochondrial T Cell ROS Generation

The tricarboxylic acid (TCA) cycle takes place in the matrix of mitochondria and is an essential component of aerobic respiration. The TCA cycle finishes the catabolism of sugar by glycolysis and converts metabolic intermediate acetyl-CoA into multiple reduced coenzymes, providing electrons to OXPHOS as well as providing a pond of essential intermediates for ATP production. Increasing evidence has surfaced supporting the claim that OXPHOS is not the sole pathway within the mitochondria that produce ROS (49–59). In the process of decarboxylation of pyruvate to acetyl-CoA, the pyruvate dehydrogenase (PDH) complex is able to produce high levels of ROS. Inhibition of PDH leads to a decrease in the generation of ROS (54, 55). α-ketoglutarate dehydrogenase complex (α-KGDH), a TCA cycle enzyme, also plays a role in ROS creation (56, 57) with its reduced form generating superoxide and hydrogen peroxide (58, 59). Lastly, succinate-driven RET causes succinate accumulation and further induces ROS generation, with the inverse catalysis of succinate dehydrogenase (SDH) (49–53). In brief summary, three steps in the TCA cycle generate ROS, pyruvate to acetyl-CoA, α-ketoglutarate to succinyl-CoA, and fumarate to succinate.

### NOXs as the Main Source of Cytosolic ROS

Besides the mitochondria, NOXs transport electrons and produce cytosolic ROS, which is essential for T cell activities (35). There are seven isoforms of superoxide-generating enzyme NOXs: NOX1-5, DUOX1, and DUOX2 (60, 61). Deficient mouse models targeting GP91phox and NOX inhibitory compounds, shown in **Table 1**, are used to study T cell NOXs (62, 75, 76). In addition, it has been ascertained that NOX2 (GP91phox) is the main isoform in T cells (60, 74). Deficiency of NOX2 in Treg cells results in an increased number of Treg cells in the heart and vessels as well as and driving to a more anti-inflammatory phenotype, with an increased expression of IL-10 and decreased expression of IL-17 (76).

**Table 1:** Inhibitory compounds targeting mtROS and cytosolic ROS (cROS) in the review.

Drugs	Detected species	Principle and targeted system	Reference
**DPI** **(Diphenyleneiodonium chloride)**	Intra-ROS, mtROS	Inhibitor of flavoenzymes, includes NOXs	(32, 38, 60–66)
**VAS2970/VAS2870**	cROS	Inhibitor of NOXs (NOX2>NOX1>NOX5>>NOX4)	(32, 39, 60, 61)
**MitoQ (Mitoquinone), MitoVitE** **MitoTEMPO**	mtROS	Mitochondria-targeted antioxidant by attaching a hydrophobic cation	(39, 67)
**NAC (N-Acetylcysteine)**	cROS	ROS scavenger, break thiolated proteins and release free thiols	(66–72)
**Catalase**	Intra-ROS	ROS scavenger, hydrogen peroxide is decomposed to water and oxygen	(64)
**Trolox**	Intra-ROS	ROS scavenger, water-soluble analog of vitamin E	(64)
**Apocynin/Diapocynin**	cROS	Inhibits the assembly of NOXs (selectivity controversies)	(60, 61, 68)
**Gp91ds-tat**	cROS	A selective NOXs peptide inhibitor	(61, 64)
**2-Acetylphenothiazine (ML171)**	cROS	NOX1 specific inhibitor, (NOX>NOX4=NOX5)	(61)
**Rotenone**	mtROS	Inhibitor of mitochondrial complex I	(5, 34, 58, 73)
**Antimycin**	mtROS	Inhibitor of mitochondrial complex III	(58)
**Pyrazolopyridine derivative (GKT136901/GKT831)**	cROS	NOX1>NOX4=NOX5	(61)
**Dihydroethidium (DHE)**	cROS	Superoxide indicator	(5, 30, 35, 38, 65–67)
**GlucoxBiotech compound M13**	cROS	NOX4>>NOX1	(61)
**ML090** **(5,12- 83 Dihydroquinoxalino(2,3B)quinoxaline)**	cROS	NOX5=NOX1=NOX4>NOX2	(61)
**GSK** **(N-(1-isopropyl-3-(1-methylindolin-6-yl)-1H-pyrrolo[2,3-b]pyridin-4-yl)-1-methyl-1H-pyrazole-3-sulfonamide)**	extracellular ROS & intracellular ROS	Inhibitor of cytochrome b558–containing phagocyte oxidase, and targeting NOX	(64)
**CPI-613**	mtROS	PDH inhibitor	(55)
**Metformin**	mtROS	Inhibitor of mitochondrial complex I	(54, 74)

### T Cell Development in the Thymus Is Affected by ROS

The thymus allows thymocytes to develop, mature, and expand by providing a distinctive microenvironment. Studying the thymic microenvironment may allow researchers to develop drugs targeting parathymic syndromes and thymus-related diseases, including myasthenia gravis (MG), pure red cell aplasia (PRCA), and hypogammaglobulinemia (77). Little is known as to how ROS might regulate T cell development within the thymus. There are some studies applying extracellular ROS to observe how ROS affects T cell development (60). The production of CD3 T cells in the thymus could be inhibited by hyperbaric oxygen *in vivo*, a treatment for tumors (60). An increased level of thymocyte survival and enhanced expression of TNF-α and IL-2 are observed with the treatment of *Ganoderma atrum polysaccharide* (PSG-1) by ameliorating ROS generation in immunosuppressed mice (78). Increasing ROS generation results in an increasing level of double-negative (DN) cells and declining levels of CD4 and CD8 single-positive (SP) cells in the thymus and further induces apoptosis in manganese superoxide dismutase 2 (SOD2) deficient mice (30). These results indicate that a moderate amount of ROS generation potentially influences thymic development.

## ROS Impacts T Cell Activation

TCR signaling pathways are affected by ROS, which trigger several proximal and distal signaling pathways in T cells (31, 79). There are studies showing that TCR stimulation induces the generation of an enormous amount of ROS, thus resulting in the activation of transcription factors nuclear factor of activated T cells (NFAT), activator protein-1 (AP-1), and nuclear factor kappa light chain enhancer of activated B cells (NF-kB) (80).

It is well established that mitochondrial ROS (mtROS) are associated with T cell activation. Mitochondrial ROS production enhances NFAT activation, leading to the induction of the transcription factor MYC (31, 79, 81). The generation of mtROS from mitochondrial complex I also induces the NFAT complex, the subunit transcription factor c-Jun in AP-1, and further increases the expression of IL-2 and IL-4 (73, 81). However, a conflicting study demonstrated that high levels of intracellular ROS, triggering the antioxidant glutathione to respond, leads to the inhibition of NFAT activation and the reduction of Myc expression (82). In addition, mtROS production, independent from complex III, has been seen to induce NF-kB and subsequently release IL-2 and IL-8 (31, 80).

NADPH oxidases (NOX) are essential enzyme complexes in the rapid generation of ROS upon T cell activation (9, 32, 38, 83–85). Inhibition of NADPH oxidase by the use of pharmacological compounds, such as diphenyleneiodonium chloride (DPI), apocynin1, and other antioxidants and inhibitors (shown in **Table 1**) results in a defective production of ROS. It has been revealed that NOX induces rapid generation of ROS, which then activates c-Jun N-terminal kinase (JNK) and NF-kB signaling, prompting an increase of IFN-γ and CD39 expression (32). However, contradictory results have surfaced where upon TCR activation the production of ROS is NADPH oxidase independent (31).

## T Cell Differentiation Impacted by ROS

Multiple studies use numerous ROS inhibitors (shown in **Table 1**) or knockout mice to test the impact of mtROS and cytosolic ROS on T cells. Autoreactive CD4 T cells deficient in NOX-derived superoxide exhibited high levels of Th1 cytokine expression (86). When there is a copious amount of superoxide, Th1 cytokines and proinflammatory chemokines revert to normal levels *via* a decreasing IL-12Rβ2 expression and P-STAT4 activation (86). In addition, ROS showed an inhibitory effect on the levels of IFN-γ and T-bet expression as well as an enhancing effect on IL-4 expression, *via* ERK1/2 signaling, for *in vitro* murine Th1 cells (68, 87). Inhibition of Nox2 using GP91phox (Nox2)-deficient mice prompted an increase in mtROS generation, elevated Th2 differentiation, and enhanced Th2 cytokines: IL-4, IL-5, and IL-13 (68, 88, 89). As for Th9 cells, there is a review that has shown that SIRT1 and HIF1α modulate both Th9 differentiation and ROS generation. However, there is no current definitive link demonstrating how ROS impacts Th9 cells (90).

Th17 cells play a critical role in protecting against extracellular pathogens. Dysregulated Th17 cells and aberrant Th1 cells, either alone or together, are associated with inflammation in autoimmune diseases (87, 91). T cells that will differentiate into Th17 require moderate levels of ROS from either mitochondria or nitro-oxidative pathways (67, 92–95). Resveratrol, a plant phytoalexin, upregulates superoxide dismutase (SOD) within mitochondria, which modulates oxidative stress and leads to Th17 differentiation (92). A study conducted by Zhi has shown that MitoQ inhibits immediate early response geneX-1 (IEX-1) knockout T cells from differentiating to Th17 cells, but wild-type (WT) T cells show no effect when exposed to MitoQ (92). This study used a broader antioxidant, N-Acetylcysteine (NAC), and found that a non-specific antioxidant blocks both WT and IEX-1 KO T cells from differentiating into Th17 cells (92). It is suggested that mtROS is required for Th17 cell generation, especially for IEX-1 knockout T cells. The importance of mtROS has been illustrated in a recent paper showing that Th17 cell generation decreases when mtROS is inhibited by MitoQ (67). High glucose levels induce the formulation of mtROS, specifically mitochondrial superoxide production, in T cells, leading to TGF-β activation and Th17 cell differentiation (67). However, there are some dissenting opinions on how ROS impacts Th17 cell differentiation (87, 91). Treatment with ROS scavenger NAC, leading to a reduced level of ROS, augment differentiation of T cell to Th17 cells (91). Another study conducted by Abimannan has found that when treating the cells with pro-oxidant, PB (5-hydroxy-2-methyl-1, 4-naphthoquinone) and H_2_O_2_, the frequency of Th17 cells had been reduced in a dose-dependent manner (87). These two studies have demonstrated that the accumulation of ROS limits Th17 differentiation. Further investigation on ROS-regulated Th17 cell differentiation and ROS-mediated inflammatory response may improve the development of treatments for inflammatory and autoimmune diseases.

Treg cells play a critical role in cancer immunology, and an imbalance of Treg cells and Th17 cells leads to autoimmune disorders. ROS generation results in Treg cell-mediated immunosuppression and limits anti-tumor T cell response in the tumor microenvironment (69). Reduced ROS generation impairs Treg function and differentiation, and it is suggested that ROS is required for the suppressive function of Treg cells (69, 96). In an exogenous H_2_O_2_ environment, Treg cells are more resistant to oxidative-induced death compared to Teff cells and memory T cells (97). ROS generation is found to be greater in Treg cells in comparison to Teff cells (96). Excessive ROS levels have been observed in aged Treg cells from >18-month-year-old mice, whereas young Treg cells exhibit controlled ROS generation, normal proliferation, and controlled inflammation (96). Limitation of ROS generation by ROS scavenger NAC has been shown to promote proliferation and survival in aged Treg cells (96). Henceforth, the phenotypic connection between ROS generation and Treg cell differentiation is found. Future research may help us to unveil how the ROS-mediated mechanism underlying Treg cells’ differentiation and function.

## T Cell Death Impacted by ROS

**Figure 3:**
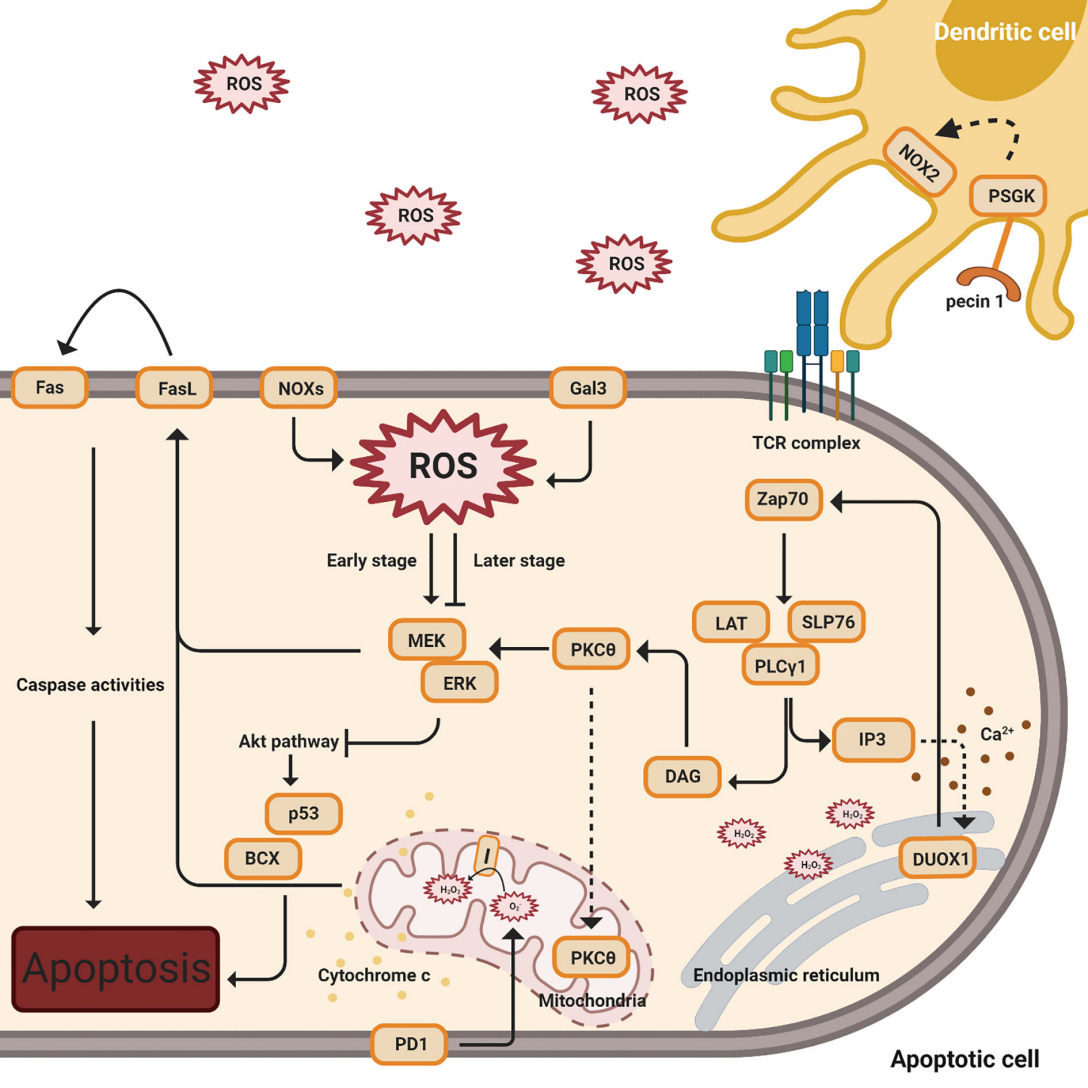
ROS regulation of activation-induced T cell death (AICD). This figure indicates multiple signal pathways involved in T cell death, including PD1 (below), Gal3, NOX2, and FasL-mediated (above) pathways. The orange color denotes protein complexes. Black solid arrows indicate the products and interactions of these pathways. The early stages denote TCR activation while the later stages indicate apoptosis. The dashed arrow affiliated with the PKCθ protein indicates translocation from the cytosol to the mitochondria. Cytochrome c release from the mitochondria to the cytosol is shown by yellow dots. The dashed line associated with IP3 designates the induction of calcium release (shown in brown dots) and the complexes’ impact on DUOX1 in the endoplasmic reticulum.

Adequate levels of intracellular ROS, including NOX-derived ROS and mtROS, emerge to regulate activation-induced T cell death (AICD) by affecting the Fas ligand (FasL) and ERK-mediated pathways (**Figure 3**) (35, 63–65, 70, 74, 98).

Following TCR stimulation, zeta chain-associated protein kinase 70 (ZAP70) is activated and phosphorylated (74, 99). ZAP70 phosphorylates the adaptor, linker of activated T cells (LAT) with the coupled recruitment of phospholipase Cγ1 (PLCγ1) and further generation of inositol 3,4,5-triphosphate (IP3) and diacylglycerol (DAG) (74, 99). IP3 binds to its receptor, IP3R1, resulting in a low concentration of calcium releasing into the ER, and this action induces Duox1, an isoform of NOX, to produce intracellular hydrogen peroxide (35). Intracellular H2O2, from NOX2 and NOX4, creates a positive feedback loop to enhance TCR signaling during T cell activation. While in later stages, as the cells undergo apoptosis, ginseng pectins selectively inhibit ERK activation, a part of the galectin-3 (Gal3) triggered pathways (35, 63, 65, 66, 70). An interesting study by Zhao has shown that walnut polyphenol extract (WPE) reduces ROS generation, decreasing the expressions of apoptosis-associated proteins Bax and p53 (98).

Separately, protein kinase cθ (PKCθ) is activated by DAG and translocates into the mitochondria, impacting the production of hydrogen peroxide from mitochondrial complex I (74). These proximal signaling eventually results in the induction of FasL (CD95) expression, a crucial signal for the induction of activation-induced cell death (AICD) (74). In addition, AICD, followed by the activation of FasL, is dependent on superoxide but not hydrogen peroxide (65).

Programmed death-1(PD-1) has an impact on ROS, independent of NOX, with a concomitant in the T cell apoptosis pathway (71). ROS levels have no impact in PD-1 low cells, while lower levels of ROS have been observed in PD-1 high cells when neither PD-1 nor PDL1 expression is blocked (71). In this study by Tkachev, they also investigated which ROS sources are affected by PD1 and demonstrated that PD1 regulates two sources of ROS, mitochondrial H_2_O_2_ and ROS upon FAO (71).

Excess effector T cells that have undergone apoptosis require removal by macrophages after a period of infection in order to conserve energy (64). The clearance of overreacted and apoptotic T cells is essential to purge in order to prevent autoimmune diseases. ROS generation is detected when dectin-1 on dendritic cells binds to the annexins on apoptotic cells (64).

## Concluding Remarks

Metabolic reprogramming of T cells is intertwined with T cell survival and proliferation. Although there have been numerous studies in the field, it is still unclear why different T cells’ subsets modulate distinct metabolic pathways: pentose phosphate pathway, glutaminolysis, aerobic glycolysis, OXPHOS, and FAO. Understanding T cell metabolic reprogramming is critical for future drug and clinical developments concerning immunological disease. It has been acknowledged for decades that ROS is generated as a byproduct during oxidative metabolism. While recent discoveries have demonstrated that low and moderate levels of ROS generated from mitochondria and NOXs are imperative in signaling T cell immunity, excess amounts of ROS result in mutation and cell damage. ROS production from oxidative phosphorylation had been studied decades prior, but there is emerging evidence that has shown multiple steps in the TCA cycle could also generate oxidative species. Both NOX-derived ROS and mtROS exhibit essential roles in the regulation of thymic development, T cell activation, T cell differentiation, and activation-induced T cell death. Such knowledge may help to reveal the impact of intracellular ROS on T cell immune response. Targeting the redox state in various T cell subsets by altering ROS could be a potential way to improve novel therapeutic strategies for treating immunological disorders. Further investigations are expected to elucidate the molecular mechanism of how ROS impacts T cell fate, metabolism, and function, with the inevitable goal being the illustration of possible novel therapies with the application of ROS scavengers in treating ROS-related diseases.

## Author Contributions

H-YP and JS contributed to the writing of the manuscript. JL, DB, JD, AK, LW, YR, and XX provided the editing of the manuscript. All authors contributed to the article and approved the submitted version.

## Funding

This work was supported by the National Institute of Health Grant R01AI121180, R21AI128325, and R01CA221867 to JS.

## Conflict of Interest

The authors declare that the research was conducted in the absence of any commercial or financial relationships that could be construed as a potential conflict of interest.
